# Integrating family planning services into HIV care: use of a point-of-care electronic medical record system in Lilongwe, Malawi

**DOI:** 10.1080/16549716.2017.1383724

**Published:** 2017-10-17

**Authors:** Hannock Tweya, Caryl Feldacker, Lisa B. Haddad, Chimango Munthali, Mwatha Bwanali, Colin Speight, Layout G. Kachere, Petros Tembo, Sam Phiri

**Affiliations:** ^a^ Center for Operational Research, The International Union Against Tuberculosis and Lung Disease, Paris, France; ^b^ Monitoring and Evaluation Department, Lighthouse Trust, Lilongwe, Malawi; ^c^ International Training and Education Center for Health, University of Washington, Seattle, WA, USA; ^d^ Department of and Obstetrics, Emory University School of Medicine, Atlanta, GA, USA; ^e^ Department of Informatics, Baobab Health Trust, Lilongwe, Malawi; ^f^ Department of Medicine, University of North Carolina School of Medicine, Chapel Hill, NC, USA; ^g^ Lilongwe, Department of Public Health, College of Medicine, School of Public Health and Family Medicine, University of Malawi, Malawi

**Keywords:** Family planning services, integration, contraceptives, electronic medical record system, antiretroviral therapy, Malawi, sub-Saharan Africa

## Abstract

**Background**: Integrating family planning (FP) services into human immunodeficiency virus (HIV) clinical care helps improve access to contraceptives for women living with HIV. However, high patient volumes may limit providers’ ability to counsel women about pregnancy risks and contraceptive options.

**Objectives**: To assess trends in the use of contraceptive methods after implementing an  electronic medical record (EMR) system with FP questions and determine the reasons for non-use of contraceptives among women of reproductive age (15–49 years) receiving antiretroviral therapy (ART) at the Martin Preuss Center clinic in Malawi.

**Methods**: In February 2012, two FP questions were incorporated into the ART EMR system (*initial* FP EMR module) to prompt providers to offer contraceptives to women. In July 2013, additional questions were added to the FP EMR module (*enhanced* FP EMR) to prompt providers to assess risks of unintended pregnancies, solicit reasons for non-use of contraceptives and offer contraceptives to non-pregnant women . We conducted a retrospective, longitudinal cohort study using the EMR routinely collected data. The primary outcome was the use of any modern contraceptive method. Descriptive statistics were used to describe the study population and report trends in contraceptive use during the initial and enhanced study periods.

**Results**: Between February 2012 and December 2016, in HIV clinics, 20,253 women of reproductive age received ART, resulting in 163,325 clinic visits observations. The proportion of women using contraceptives increased significantly from 18% to 39% between February 2012 and June 2013, and from 39% to 67% between July 2013 and December 2016 (chi-square for trend p < 0.001). Common reasons reported for the non-use of contraceptives among those at risk of unintended pregnancy were: pregnancy ambivalence (n = 234, 51%) and never thought about it (n = 133, 29%).

**Conclusion**: Incorporating the FP EMR module into HIV clinical care prompted healthcare workers to encourage the use of contraceptives.

## Background

The scale-up of antiretroviral therapy (ART) for people living with human immunodeficiency virus (PLHIV) in sub-Saharan Africa (SSA) is one of the most remarkable achievements in public health in the past decade. In 2015, approximately 12.1 million PLHIV were on ART in the region [1]. The availability of ART has considerably decreased mortality and morbidity and improved quality of life [2,3]. This, together with the cultural significance of parenthood, may be reason for the increased fertility rates that have been observed in populations of women on ART in several SSA settings [4–6]. Helping women living with HIV achieve their family planning (FP) intentions is an important public health objective in SSA.

SSA has the lowest rate of contraceptive use in the world; only 28% of married women in SSA used modern contraceptive methods in 2015 [7]. The fertility rate in Malawi is 4.4 per woman and is one of the highest in SSA. Yet, it is estimated that 41% of pregnancies in Malawi are unintended [8]. The prevention of unintended pregnancy is a critical public health issue for HIV-positive women. There are significant consequences such as HIV-related maternal morbidity [9], the vertical transmission of HIV [10], mental health problems [11], and financial and social costs. In SSA, FP services are mostly provided in clinics that are not connected to HIV clinics. However, a growing body of evidence suggests that integrating FP services into HIV care can be a way of increasing contraceptive use among HIV-positive women[12–14].

In 2010, the Lighthouse Trust in Malawi piloted the integration of FP services into routine HIV care at the Lighthouse Clinic. A wide range of modern contraceptives were offered to HIV positive women. The pilot demonstrated that the integration of FP services into HIV care is achievable, is well received by women on ART and can lead to an increased uptake of contraceptives [15]. In 2011, the Malawi HIV programme integrated FP services into HIV clinical care in all ART facilities [16]. Healthcare providers were encouraged to routinely provide condoms to all adults receiving HIV care and offer all women, as a minimum, an injectable contraceptive, Depot medroxyprogesterone Acetate, (DMPA). In 2016, 23% of women on ART in Malawi were on DMPA provided within the country’s ART clinics [17].

Although the coordinated integration of FP services into HIV services is starting to occur, health system strengthening is challenged by inadequate enforcement of policies and guidelines, limited resources and funding, and a lack of leadership [18,19]. In addition, providers in HIV settings faced with high patient numbers often have limited time to counsel women about pregnancy risks and contraceptive options. Some women are therefore unable to access available FP services in HIV clinics. Ensuring that women receive comprehensive FP services remains a critical challenge for HIV clinical care.

One innovative means of addressing FP integration in HIV care is user-friendly, point-of-care (POC) electronic medical record (EMR) systems; A POC EMR system is a digital version of a paper chart that combines data collection and patient care processes simultaneously, making both the past and current medical history of the patient available to the provider. EMR systems can improve healthcare by increasing adherence to therapeutic guidelines and protocols, informing clinical decisions and limiting medication errors [20,21]. The use of POC EMR systems in HIV care can offer opportunities to improve adherence to FP guidelines and ensure that women of reproductive age (15–49 years) are offered FP services when they receive their HIV clinical care. This paper: describes the use of EMR systems to support healthcare providers to better meet the FP needs of HIV-infected women of reproductive age; investigates trends in contraceptive use among these women; and explores some of the reasons for the non-use of contraceptives among women at a high volume HIV clinic in Lilongwe, Malawi.

## Methods

### Study design and setting

We conducted a retrospective, longitudinal, cohort analysis of 20,253 HIV-positive women aged 15–49 who accessed ART services between 17 February 2012 and 31 December 2016, at a public ART clinic, the Martin Preuss Centre (MPC) at Bwaila Hospital situated in urban Lilongwe, Malawi. During this time, there were 163,325 clinic visits among these women.

The Lighthouse Trust operates two public ART clinics, the MPC and the Lighthouse Clinic. These are described in detail elsewhere [22]. In brief, both clinics are located in Malawi’s capital, Lilongwe. The clinics use an ART EMR system for the clinical management of ART patients. The EMR system was developed by Baobab Health Trust, a local non-governmental organization that leads the development of EMR systems in Malawi in collaboration with the Lighthouse Trust. The ART EMR is real-time, POC, intranet-based software that employs the MySQL OpenMRS data model. The EMR system uses touchscreen technology with terminals in reception and clinical consultation rooms [23]. Key EMR system components include the registration of demographics and anthropometric measurements, WHO HIV clinical staging, tuberculosis screening, record of pregnancy status, clinical review, drug dispensing, and the management of laboratory samples and test results (CD4 count, viral load, etc.). Data management is now entirely based on the EMR system resulting in near complete patient level data [23]. The system also allows documentation of co-morbidities and infections such as hypertension and cancer. ART visits for new patients are scheduled monthly for the first six months on ART, and every two or three months thereafter if no clinical complications occur. ART programme outcomes (alive and on ART, lost to follow-up (LTFU), stopped ART, dead or transferred-out) are updated in EMRs at each clinic visit or retrospectively. LTFU is defined as failure to come to the clinic for at least 60 days from the patient’s appointment date.

Although integrated FP services were piloted at Lighthouse Clinic in 2010, the FP questions were not incorporated in the EMR until July 2014.

Data for this analysis were obtained from the POC EMR system at MPC. In December 2016, the MPC had approximately 22,000 ART patients in care (10,887 or 49% of women of reproductive age), There were, on average, 450 attendances each day.

### Integration of FP services at MPC

Following the implementation of the 2011 Malawi ART/Prevention of Mother-to-Child Transmission of HIV guidelines, the MPC integrated FP services into HIV clinical care. Healthcare providers routinely offered condoms and DMPA to women. Initially, some contraceptive methods were not offered at MPC because of its location adjacent to a family health unit [15]. Women who preferred other contraceptives were referred to this unit which was just 60 meters away, in the grounds of Bwaila Hospital. On 17 February 2012, the Lighthouse Trust, in partnership with Baobab Health Trust, incorporated two FP questions (*initial* FP EMR module) into the ART EMR. The initial FP EMR module prompted providers to ask women about their contraceptive use and methods. In July 2013, the initial FP EMR module was enhanced by prompting providers to assess non-pregnant women for risks of unintended pregnancy, solicit reasons for the non-use of contraceptives, counsel patients, and offer condoms and DMPA to women. Women who preferred other types of contraceptives were referred to the family health unit. Possible self-reported reasons for contraceptive non-use included: partner’s wish, religion, fear of side effects, never thought about it and uninterested. Women were classified as ‘not at risk’ of unintended pregnancy if they were not sexually active, wanted to become pregnant or did not need contraceptives because of medical reasons (e.g. hysterectomy). The enhanced FP EMR separately prompted health providers to offer condoms during the consultation timeframe. In February 2015, FP services were fully integrated at MPC. Providers were trained in FP services and a wide range of long-acting reversible contraceptive (LARC) methods were offered within the ART clinic. These methods included oral contraceptive pills (OCPs), DMPA, copper intrauterine devices (IUDs) and contraceptive implants. From that point forwards, patients were informed about the availability of FP services through both oral and visual methods such as flip charts and films.

### Data management and variables

Clinical and other data for all women, aged 15–49 years, who accessed ART between 17 February 2012 and 31 December 2016 were extracted from the EMR system. The following variables were generated from the EMR: age, clinic visit date, pregnancy status, contraceptive use (yes or no), names of contraceptive methods, offered contraceptive methods, offered dual contraceptive methods, reasons for not using contraceptive methods, ART programme outcome and outcome date. Information on marital status was not included as this is not captured in the EMR.

For the purposes of the analysis, each woman’s index visit was identified within three monthscalendar quarter. A visit close to the middle of each quarter was selected as the woman’s index visit. The implementation of the integrated FP module was in two stages: 1) the initial FP EMR module (February 2012 to June 2013), and 2) the enhanced FP EMR module (July 2013 to December 2016) which includes the period of more comprehensive integration of FP services from February 2015. Women who were given ART during both of these periods were included in the analysis that covered both stages.

Contraceptive methods were categorized as: 1) implants; 2) DMPA; 3) OCPs; 4) other highly effective contraceptives (IUDs, tubal ligation and vasectomy); and 5) less effective contraceptive methods (female/male condoms and traditional methods). Dual contraceptive methods were defined as condoms plus any other effective contraceptive methods recommended by the World Health Organization (e.g. sterilization, hormonal methods (implant, OCPs, DMPA), IUDs) [24].

### Statistical analysis

The primary outcome was contraceptive use, which was defined as the self-reported use of any modern contraceptive method during the index visit. We calculated the proportion of contraceptive use as the number of women reported using contraceptives at the time of their ART clinic visit over the total number of non-pregnant women who accessed ART care during each quarter. The denominator included some women who were not sexually active because 1) sexual activity status may change within a short time and 2) women might have falsely reported not being sexually active due to the sensitivity of discussing their sexual life.

Descriptive statistics were undertaken. They included the median and interquartile range (IQR) for the women’s age, and frequencies for categorical variables (proportions of contraceptive use, contraceptive assessment, contraceptive methods, reasons for non-use, etc.). We analysed information reported as reasons for contraceptive non-use at the time of the first FP review during the implementation of the enhanced EMR FP module. We acknowledge that reasons for non-use might change over time. The chi-square test for trend was used to test for statistically significant change in the proportion of women using contraceptives over time.

## Results

**Figure 1.**
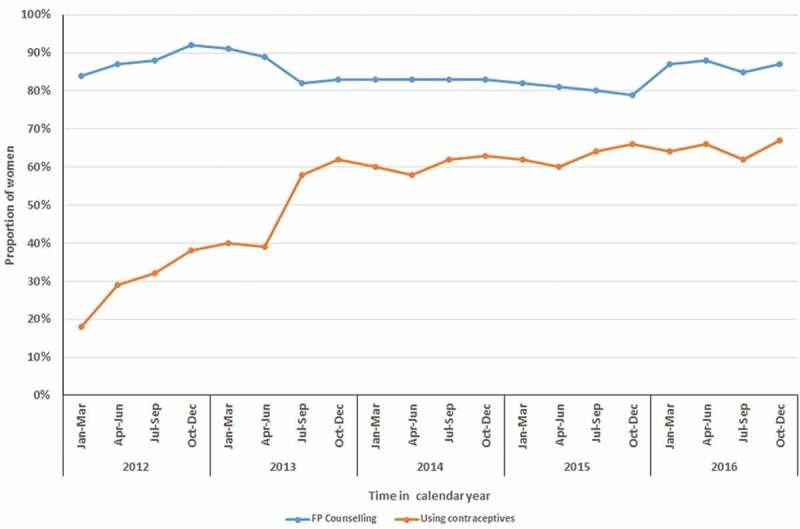
Contraceptive use among HIV-positive women of reproductive age accessing antiretroviral therapy in Lilongwe, Malawi (N = 20,253).

A total of 20,253 women had 163,325 clinic visits between 17 February 2012 and 31 December 2016. At 31 December 2016, 11,648 women (57%) were alive and on ART, 3370 (17%) had transferred their ART care to other facilities, 245 (1%) were deceased, 228 had stopped ART and 4762 (24%) were LTFU. A total of 9460 women received ART during the implementation of initial FP EMR module and 18,013 women received ART during the implementation of the enhanced EMR FP module. On average, information on contraceptive use was not available for 16% of the clinic visits. The median age of the women was 30 (IQR: 25–35) years at first review. Between February 2012 and June 2013, the use of contraceptives increased significantly from 18% to 39% during implementation of the initial FP EMR module, and from 39% to 67% during the implementation of the enhanced FP EMR module (chi-square for trend p < 0.001) (Figure 1).

**Figure 2.**
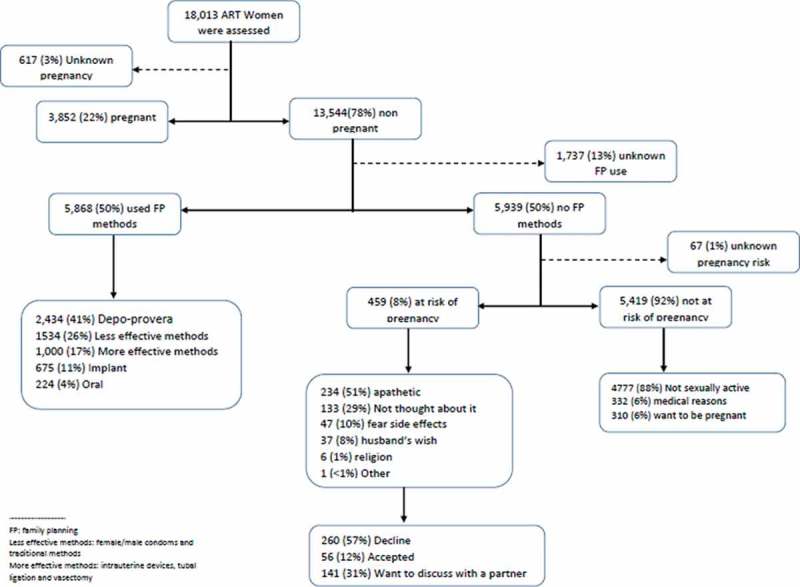
Flow diagram of contraceptive use and reasons for the not using on the first FP review during the implementation of the enhanced EMR FP module between July 2013 and December 2016, at Martin Preuss Centre, Lilongwe, Malawi.

Figure 2 presents the flow diagram of contraceptive use and reasons for non-use at the first FP review during the implementation of the enhanced EMR FP module. Only the enhanced EMR module prompted providers to ascertain their reasons for non-use. Of the 18,013 women who accessed ART services during the enhanced FP EMR module, 3852 (22%) were pregnant and pregnancy status was unknown in 617 (3%). Of the 13,544 non-pregnant women, use of contraceptives was unknown in 1737 (13%) of women. Out of the remaining 11,807 women, 5870 (50%) were using FP contraceptive methods. Of these 41% used DMPA, 26% used less effective methods, 17% used other highly effective methods, 11% had implants and 4% used OCPs. Of the 5,939 women who were not using contraceptives, the risk of unintended pregnancy was not established in 61(1%) women. Of the remaining 5878 women, 459 (8%) were found to be at risk of unintended pregnancy and 5419 (92%) not at risk. Of those who were not at risk of becoming pregnant, 310 (6%) reported that they wanted to become pregnant, 332 (6%) had medical reasons for not requiring contraceptives, and 4767 (88%) reported that they were not sexually active. All women who were at risk of unintended pregnancy during the implementation of the enhanced FP EMR period were asked to give reasons for not using contraceptives. Reasons given included: pregnancy ambivalence [n = 234, 51%], never thought about it [n = 133, 29%], fear of side effects [n = 47, 10%], and husband’s wish [n = 37, 8%]. Women who were at risk of unintended pregnancy were offered contraceptives: 56 (12%) women started using contraceptives on the same day, 141 (31%) wanted to seek their partner’s approval and 261 (57%) declined. At six-month follow-up, 339 (74%) of the 459 at risk women were still in care and 18 (5%) of these women were at risk of unintended pregnancy.

The use of more effective contraceptives increased from 69% to 78%, during the study period largely because of the increase in the use of IUD, tubal ligation and vasectomy (chi-square for trend, p < 0.001) (Table 1). DMPA was the most common contraceptive method (53%). The use of OCPs remained constant at 5%. The proportion of dual contraceptive method use remained low over time (< 10%) while the proportion of less effective methods declined from 31% to 22%.

**Table 1.** Frequency and percentage distribution of ART women aged 15 − 49, by contraceptive use and method in selected calendar quarters, 2012 to 2016.

	First Phase				Second Phase					
Year	2012		2013		2014		2015		2016	
Indicators/Months	Jan-Mar	Jul-Sep	Jan-Mar	Jul-Sep	Jan-Mar	Jul-Sep	Jan-Mar	Jul-Sep	Jan-Mar	Jul-Sep
All ART women of reproductive age	4,233	6,569	6,971	7,230	7,674	8,280	8,856	9,380	9,841	10,528
Contraceptive assessment	3,424 (81%)	5,440 (83%)	6,015 (86%)	5,893 (82%)	6,387 (83%)	6,856 (83%)	7,292 (82%)	7,529 (80%)	8,518 (87%)	8,930 (85%)
Using contraceptive^¥^										
Yes	616 (18%)	1,742 (32%)	2,392 (40%)	3,449 (59%)	3,898 (61%)	4284 (62%)	4,549 (62%)	4,866 (65%)	5,485 64%)	5,580 (62%)
No	2,808 (82%)	3,698 (68%)	3,623 (60%)	2,444 (41%)	2,489 (39%)	2,572 (38%)	2,743 (38%)	2,663 (35%)	3,034 (36%)	3,350 (38%)
Contraceptive methods										
Highly effective methods	422 (69%)	1,270 (73%)	1,719 (72%)	2,445 (71%)	2,884 (74%)	3,132 (73%)	3,502 (77%)	3,882 (80%)	4,286 (78%)	4,370 (78%)
Implant	54 (13%)	154 (12%)	215 (13%)	304 (12%)	361 (13%)	384 (12%)	412 (12%)	424 (11%)	424 (10%)	406 (9%)
DMPA	249 (59%)	694 (55%)	891 (52%)	1,381 (56%)	1,491 (52%)	1,583 (50%)	1,812 (52%)	2,017 (52%)	2,280 (53%)	2,308 (53%)
Oral	28 (7%)	67 (5%)	78 (5%)	131 (5%)	158 (5%)	168 (5%)	175 (5%)	180 (5%)	232 (5%)	219 (5%)
Intrauterine devices, tubal ligation and vasectomy	91 (22%)	355 (28%)	535 (31%)	629 (26%)	874 (30%)	997 (32%)	1,103 (32%)	1,261 (32%)	1,350 (32%)	1,437 (33%)
Less effective methods	194 (31%)	472 (27%)	673 (28%)	1,004 (29%)	1,015 (26%)	1,152 (27%)	1,048 (23%)	984 (20%)	1,199 (22%)	1,210 (22%)
Condoms	194 (100%)	468 (99%)	668 (99%)	995 (99%)	1,011 (99%)	1150 (99%)	1,040 (99%)	979 (99%)	1,167 (97%)	1,183 (98%)
Tradition	0	4 (1%)	5 (1%)	9 (1%)	4 (<1%)	2 (<1%)	8 (1%)	5 (1%)	32 (3%)	27 (2%)
Dual contraceptive method use^β^	-	-	-	294 (9%)	264 (7%)	262 (6%)	204 (4%)	71 (1%)	321 (6%)	323 (6%)

^¥^Chi-square for trend p < 0.001; ^β^Dual contraceptive method use was defined as use of condoms plus any other effective contraceptive methods recommended by the World Health Organization (e.g., sterilization, hormonal methods (implant, OCPs, DMPA), IUDs)

## Discussion

This study reports on the development, implementation and evaluation of an EMR-based intervention designed to prompt providers to facilitate access to contraceptives among HIV-infected women in ART care in Malawi. Contraceptive use increased significantly during the intervention and providers identified reasons for non-use that can be considered in future ART programmes that integrate FP. There was a rapid increase in contraceptive use during the implementation of the enhanced FP EMR module compared with the initial FP EMR module, possibly due to increased counselling and FP-related communication with the women.

This study demonstrates the benefits of using a FP-focused EMR in HIV clinics with high patient volume. The FP EMR helps ensure adherence to guidelines and should be included as an important strategy to successfully integrate provider-initiated FP services into HIV clinical care. Human resource shortages may inhibit integration of FP services in limited resource settings [19]. Although task shifting and decentralization merit consideration in addressing shortages of healthcare workers [25], healthcare providers in high HIV prevalence settings still struggle to cope with high ART patient volumes. The high patient volumes negatively impact on providers’ ability to address broad and diverse patient needs. In this study, contraceptive use increased from 18% to 67% during the study period. The proportion of contraceptive use observed at MPC in 2016 is higher than both Malawi’s national average of 58% [8] and the average of 42% for HIV-positive women attending Lighthouse Clinics and 29% reported by Phiri et al. [15]. The data from Phiri et al. [15] were for the period, August 2010 to February 2013, before integrated FP services were offered at MPC. Although not all increases in the observed contraceptive prevalence can be attributed exclusively to the FP EMR, the FP-based EMR helps to ensure that healthcare providers adhere to FP guidelines, including providing consistent ongoing FP counselling. These results strongly suggest that the FP-EMR prompts contribute to the observed increase in contraceptive use.

During the intervention an increase in modern contraceptives use was observed. This was largely due to an increase in highly effective contraceptives such IUDs, tubal ligation and vasectomy. These highly effective contraceptives were accessed through referral to the nearby family health clinic during the study period. Ensuring the availability of these methods is a way of helping HIV-infected women attain their fertility goals. The relatively constant use of DMPA and OCPs during the study period also suggests that women may prefer to remain with known familiar methods. However, expanding access to a broad contraceptive mix is important for providing options to women who wish to better space their pregnancies or delay childbearing.

The enhanced FP EMR module offered a structured prompt to providers for discussion of reasons for non-use with women within a short consultation timeframe. This brief counselling and education communication is repeated at each visit, and this approach may help dispel common misconceptions about contraceptives and assist women to make informed contraceptive choices. Addressing these FP issues at every visit is important because an individual’s FP needs may change. The enhanced FP EMR may also assist providers to recognize and address the full range of FP needs for ART patients within the constraints of their high clinic workloads. This enables providers to identify individuals who are planning pregnancies but also need information on safe conception practices. Unlike previous studies that evaluated reasons for non-use [26,27], the study showed that pregnancy ambivalence and ‘never thought about it’ were the most common reasons. With knowledge of contraceptive methods almost universal in Malawi [8], it was expected that indecision associated with modern contraceptive methods would not be so common a reason for non-use in this population. Moreover, almost a third of the women reported not being sexually active. While many of these women may not be at risk of unwanted pregnancy, it is possible that some women did not want to discuss their sexual life with providers. This highlights that having prompts may not be enough to ensure that FP assessment for contraceptive needs is suitable for all women. Other factors may limit the provider’s ability to address all relevant issues during the clinic consultation. Increased attention to provider–patient interactions and ensuring a sensitive enabling environment for the discussion of sensitive issues should be considered in future interventions.

Unfortunately, few women (6%) reported dual-method use, highlighting that, despite an increase in pregnancy prevention, protection from sexually transmitted infections and HIV transmission to uninfected partners is inadequate. Although these findings are not surprising [28,29], the decreasing trend in condom use both alone and in conjunction with other more effective contraceptive methods is concerning. The EMR routinely prompts health providers to offer condoms to all adults in ART clinics, yet despite these attempts, the use of condoms in combination with other contraceptives remains low. In another study in Malawi, the most common reason for the non-use of condoms was related to the male partner [30]. Women required their partner’s approval, indicating the crucial role that male partners have in reproductive choices [31]. Given the importance of condom use for dual prevention, the development of novel condom promotion strategies that take into account the role of the male partners should be promoted to encourage the use of condoms among HIV-infected women and their partners. Providers could also be encouraged to ensure ongoing counselling on HIV prevention as part of the FP-centered conversation.

### Limitations

There are some limitations. First, the ascertainment of contraceptive use was not complete despite a prompt for everyone, possibly due to downtimes in the EMR system. Information on contraceptive use was not available in 16% of the clinic visits. Further refinement to the EMR systems are required to ensure that women of reproductive age are assessed at all clinic visits and offered contraceptives if so required. Additionally, routine information on contraceptive uptake before any FP EMR intervention was not available. This impeded a comparison of contraceptive use before and after intervention. Nevertheless, the rapid increase in contraceptive use after implementation of the FP EMR module strongly suggests the positive effect of the intervention on contraceptive use. Last, men and male partners were not included in the FP EMR.We did not attempt to investigate FP issues for HIV-infected men on ART.

### Conclusions

Overall, for HIV programmes with high patient volumes, the use of EMR systems for integrated FP services may assist in prompting healthcare workers to offer contraceptives to women who need them and also improve the documentation of contraceptive usage. Despite the positive effects of the FP EMR module, few women used dual contraceptive methods. Follow-up research could further improve the benefits of the FP EMR by exploring factors that reduce dual-method uptake, examining the provider–patient relationship, and gaining improved understanding of pregnancy ambivalence among this population.

## Paper context

In high HIV prevalence settings, healthcare providers are unable to properly counsel women about contraceptive options due to high patient volumes in HIV clinics. We implemented a family planning focused, electronic medical record system in an ART clinic to ensure that women are educated and offered the contraception method of their choice. This information may help providers working in high HIV prevalence settings to more successfully integrate FP services into HIV clinical care.
